# Endocrine Disrupting Chemicals in Human Milk: A Systematic Review of Concentrations and Potential Health Implications

**DOI:** 10.1007/s40572-025-00515-5

**Published:** 2025-11-25

**Authors:** Fiona Dunn, Hannah Sullivan, Megan Romano, Christina D. Chambers, Joseph M. Braun, Katherine E. Manz

**Affiliations:** 1 MRC Epidemiology Unit, University of Cambridge, Cambridge, UK; 2 Department of Environmental Health Science, University of Michigan, Ann Arbor, MI 48109 USA; 3 Department of Epidemiology, Dartmouth Geisel School of Medicine, Lebanon, NH USA; 4 Department of Pediatrics, University of California San Diego, La Jolla, San Diego, CA 92093 USA; 5 Department of Epidemiology, Brown University School of Public Health, Providence, RI USA; 6 Clinical and Translational Research Institute, University of California San Diego, San Diego, CA USA

**Keywords:** Endocrine disrupting chemicals, Human milk, Lactation, Nursing, Exposure

## Abstract

**Purpose of Review:**

Endocrine-disrupting chemicals (EDCs) disrupt the synthesis, transport, action, or metabolism of endogenous hormones in the human body. EDCs often enter the body through inhalation, ingestion, or dermal contact and can accumulate in the body. Remobilization or transfer of EDCs can occur during lactation, causing human milk to become contaminated with a variety of EDCs, which could expose nursing infants and children to these chemicals.

**Recent Findings:**

Several studies have examined the concentration ranges for one or multiple EDC(s) in human milk. Additional studies document associations between EDC exposure and adverse health outcomes, many of which are in adult populations. It is therefore essential to understand the extent to which EDCs in human milk contribute to cumulative early-life exposures.

**Summary:**

We performed a literature review of peer-reviewed studies reporting concentrations of one or more of the following EDCs in human milk during or after 2004: bisphenols, organochlorine pesticides (OCPs), polycyclic aromatic hydrocarbons (PAHs), parabens, polybrominated diphenyl ethers (PBDEs), polychlorinated biphenyls (PCBs), per- and polyfluoroalkyl substances (PFAS), and phthalates. We identified concentration ranges for each chemical detected in human milk and health impacts associated with early-life exposures to EDCs noted across studies from this review. Determining the presence of EDCs in human milk and the associated effects of exposure through nursing is essential to develop feeding recommendations that safeguard infant and child health.

**Supplementary Information:**

The online version contains supplementary material available at 10.1007/s40572-025-00515-5.

## Introduction

The World Health Organization recommends exclusive breastfeeding for the first six months of infant life, citing human milk as the best source of nutrition for infants as protection against short- and long-term adverse health outcomes [1, 2]. Studies have linked infant consumption of human milk to lower infection rates in infancy and lifelong benefits such as decreased rates of learning disabilities, diabetes, obesity, and hypertension and increases in IQ [3]. Maternal transfer of bioactive compounds, such as microbes, immunoglobulins, and lactoferrins, is essential for the development of the neonatal gut microbiome [4]. In addition to immune and health benefits, human milk contains all of the essential proteins, carbohydrates, and fats needed for the nursing infant [5]. The lipid content of milk, in particular, is important to meet the energy requirements of the growing infant and increases with the length of lactation to account for growing energy needs [5].

**Fig. 1:**
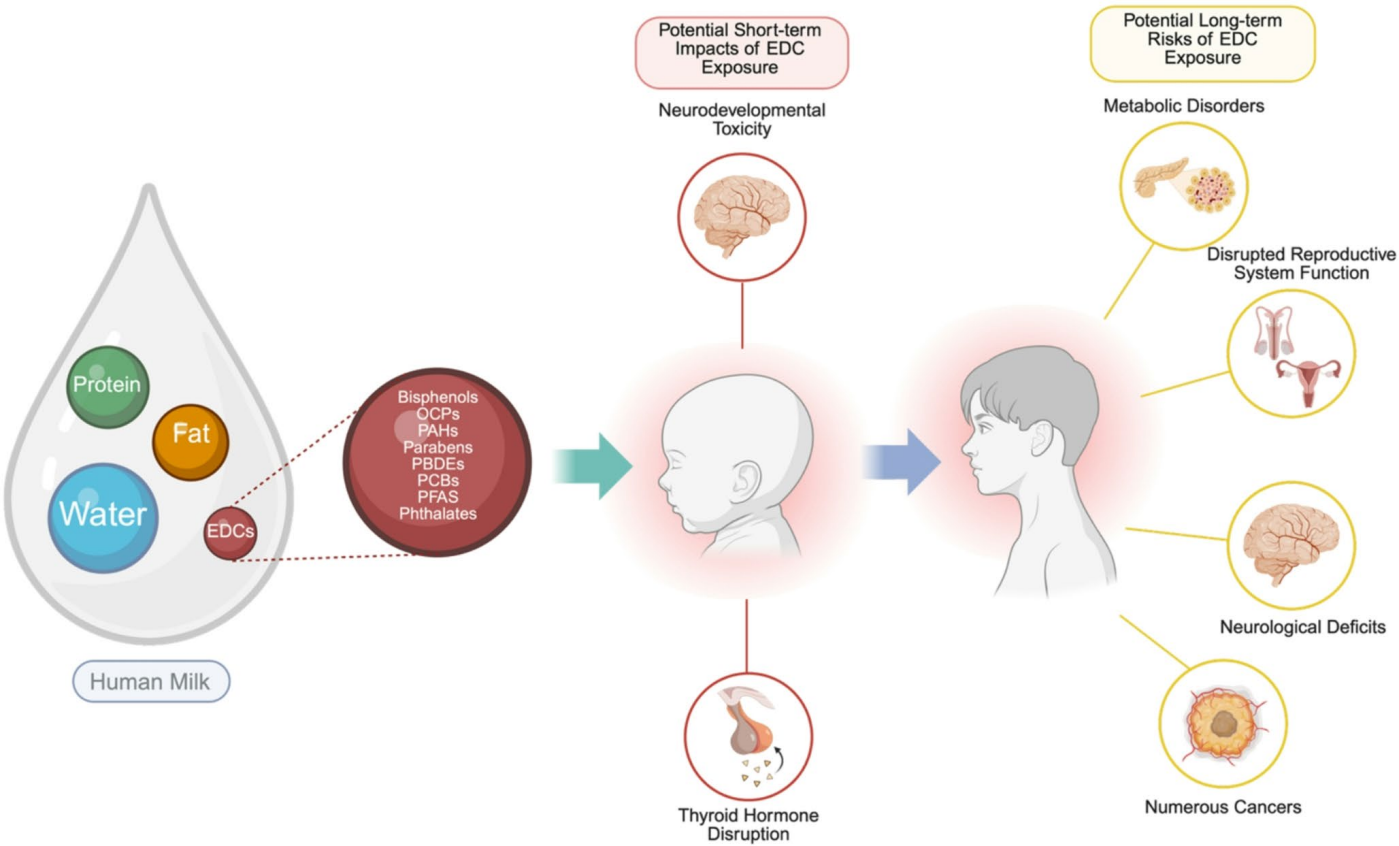
Potential impacts of neonatal exposures to EDCs, commencing with exposure via ingestion of human milk (as examined in this review). Created in BioRender. Manz, K. (2025) https://www.biorender.com/

Endocrine-disrupting chemicals (EDCs) are synthetic or naturally occurring chemicals that disrupt the normal function of hormones in the human body [6]. Anthropogenic chemicals that accumulate in the environment have been highly studied in recent decades as potential EDCs due to concerns about the health effects of exposure to EDCs [7]. EDCs may cause adverse health effects through targeted and global dysregulation of organ and organ system homeostasis [8]. Adult exposure to some EDCs increases the risk of gestational and type 2 diabetes, neurodevelopmental deficits, certain cancers, and reproductive disorders [9]. Fetal and early life exposure to EDCs may have adverse impacts on development. Exposures during childhood are associated with an increased risk of learning disabilities or deficits, mental health problems, obesity, and reproductive function [9] (Fig. 1).

To mitigate the health impacts of early life exposure to EDCs, it is essential to understand how and to what extent pre- and postnatal exposures occur. During gestation, the fetus is exposed to EDCs via placental transfer. Bisphenols, parabens, pesticides, phthalates, polychlorinated biphenyls (PCBs), and polybrominated diethyl ethers (PBDEs) have been detected in humans in either matched maternal and umbilical cord serum samples or placental samples, indicating transfer of maternal body burden of EDCs to the fetus [10]. Postnatally, studies suggest a continued risk of infant EDC exposure through contaminated human milk due to the mobilization of maternal lipid stores and passive transport of lipid- and protein-bound and water-soluble chemicals during human milk formulation, though the mechanism and extent of EDC transfer from mother to infant varies by chemical compound due to differing chemical properties [1, 11]. Previous studies indicate that levels of EDCs in human milk reflect the maternal body burden of (and thus neonatal and potentially infant and toddler exposure to) EDCs [2, 3]; further research is needed to assess chemical-specific rates transfer into human milk, as well as the risk to nursing infants [11].

This review will provide information on the extent to which EDCs are detected in human milk and document the potential risks of early-life EDC exposure. To accomplish this, we searched for studies that detected any EDC from eight pre-identified chemical classes in human milk samples. We compared the levels of EDCs detected by chemical class and discussed the geographic scope of EDCs detected in human milk, current practices, potential gaps in knowledge about the detection of EDCs in human milk, and health risks associated with neonatal, infant, and toddler exposure to EDCs.

## Methods

**Fig. 2:**
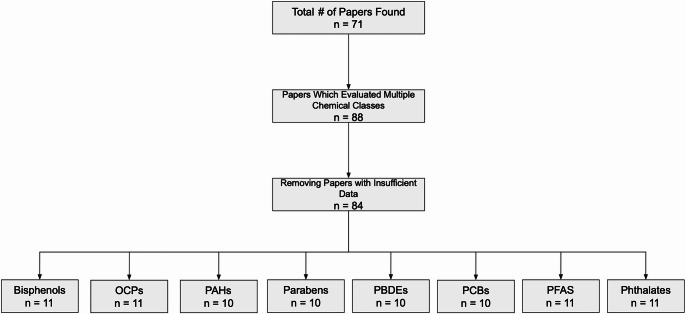
Flow chart to depict the process of finding, honing, and categorizing studies for inclusion in this review, n = no. of studies reviewed. “Adjustment for papers which evaluated multiple chemical classes” indicates that the studies which measured chemicals from multiple of our eight classes were counted separately for each chemical class

We performed a narrative literature review of research that aimed to detect EDCs in human milk. Between June and September, 2024, two reviewers utilized Google Scholar and PubMed to find peer-reviewed literature reporting human milk concentrations of EDCs. We found that the primary chemical classes detected in human milk included bisphenols, organochlorine pesticides (OCPs), polycyclic aromatic hydrocarbons (PAHs), parabens, polybrominated diphenyl ethers (PBDEs), polychlorinated biphenyls (PCBs), per- and polyfluoroalkyl substances (PFAS), and phthalates. We performed searches using combinations of keywords: “concentration,” “breast milk,” “human milk,” “bisphenols,” “BPA,” “organochlorine pesticides,” “PAHs,” “parabens,” “PBDEs,” “PCBs,” “PFAS,” “phthalates,” and “endocrine-disrupting chemicals.” We included studies that collected samples from any studies conducted during or after 2004 to ensure data were more relevant to recent trends and conditions (within the past 20 years). This review was limited to English studies only. In total, 71 studies were considered in this review (Fig. 2). All studies were verified by both reviewers to ensure accurate and comprehensive data collection.

From each study, we recorded the publication year, milk sample collection year, country of sample, chemicals detected, detection method utilized, and concentrations detected of each chemical. If provided, we also recorded the week postpartum that the milk was collected, the type of milk collected, and any noted health outcomes. We recorded the minimum, median, and maximum EDC concentrations. When this information was not available, we recorded alternative metrics and univariate statistics such as means or quartiles. We excluded studies that did not report means, quartiles, minimum, median, or maximum concentrations (Fig. 2). Limits of detection were not consistently reported in all studies; therefore, we recorded concentrations that were less than the limit of detection as 0 ng/mL or ng/g lipid, depending on the chemical class.

In this review, results are organized into a context table (Table 1) detailing the major uses, exposure routes, and health impacts for each chemical class studied. For each chemical class, further details of the pathway from exposure to chemical entry into human milk are outlined before descriptions of the results. Quantitative data on concentrations, number of samples collected, and weighted medians and maxima for all studies within each chemical class are recorded in the supplemental materials (Tables S1-8).

**Table 1:** Descriptions of the Uses of and routes of exposure to EDC classes and chemicals detected in this review. This information helps Us to understand how lifetime maternal exposures occur, which may then translate to gestational, neonatal, and infant exposure through lactation (mechanisms for early-life exposure for each chemical class discussed further in the body of the text). Of note is that exposure-related health risks are proposed in cited studies, though not all health risks have been confirmed to be associated with the exposure of interest. Note: all compounds with an * are metabolites of another parent compound within the same chemical class

EDC Class	Chemicals analyzed in this study	Uses	Common Routes of Exposure	Proposed Exposure-Related Health Risks
Bisphenols	BPA, BPAF, BPS, BPF	Used to make plastics, epoxy resins, food and beverage containers [12–14]	Ingestion, inhalation, dermal absorption [14]	Increased risk of Type 2 diabetes, adverse reproductive and developmental effects, and immune system problems [15]
OCPs	p, p-DDE, p,p-DDD, p,p-DDT, α-, β-, δ-, and γ-HCH, Oxychlordane, Cis-chlordane, Trans-Nonachlor, Dieldrin, Mirex, Cypermethrin, Heptachlor, Heptachlorepoxide, Aldrin, Endrin, Endosulfan-I and -II, Methoxychlor, Pentachlorobenzene, Hexachlorobenzene, Pentachloroanisol, Octachlorostyrene, Trans-chlordane, Cis-heptachlor, Parlar 26 toxaphen, Parlar 50 toxaphen, Chlorpyrifos-ethyl, Endosulfan sulfate, Endrin ketone, Endrin aldehyde, Cis-Chlorantraniliprole, Trans-Chlorantraniliprole, o,p-DDD, o,p-DDE, o,p-DDT	Pest control in agricultural and residential settings [16]	Ingestion, inhalation, dermal absorption [16, 17]	Endocrine, thyroid, and reproductive system disruption [18]
PAHs	Naphthalene, Acenaphthylene, Acenaphthene, Fluorene, Phenanthrene, Anthracene, Fluoranthene, Pyrene, Cyclopenta[c, d]pyrene, Benz[a]anthracene, Chrysene 5-methylchrysene, Benzo[b]fluoranthene, Benzo[k]fluoranthene, Benzo[j]fluoranthene, Benzo[a]pyrene Indeno[1,2,3-cd]pyrene, Benzo[g, h,i]perylene, Dibenz[a, h]anthracene, Benzo[b + k]fluoranthene, Benzo[c]fluorene, Benzo[b + j]fluoranthene	Byproducts of incomplete pyrolysis of organic matter, during forest fires, fossil fuel combustion, domestic cooking, smoking [19–21]	Ingestion, inhalation, dermal absorption [22].	Toxic, mutagenic, and carcinogenic effects [20]
Parabens	Methylparaben, Ethylparaben, Propylparaben, Butylparaben, Isopropylparaben, Isobutylparaben, Benzylparaben	Preservatives to prevent mold and yeast, personal care products [23]	Ingestion, dermal absorption [24]	Disrupted endocrine activity and spermatogenesis, infertility [25, 26]
PBDEs	BDE-47, BDE-99, BDE-100, BDE-153, BDE-154, BDE-183, BDE-209, BDE-15, BDE-28, BDE-196, BDE-197, BDE-206, BDE-207, BDE-49, BDE-66, BDE-85, BDE-138, BDE-118	Brominated flame retardants added to many household and commercial products, such as electronic equipment and textiles [27, 28]	Inhalation, dermal absorption [29]	Neurodevelopmental deficits, reproductive system disruption, and thyroid dysregulation [27–30]
PCBs	PCB 18, PCB 28, PCB 37, PCB 44, PCB 49, PCB 52, PCB 66, PCB 70, PCB 74, PCB 77, PCB 81, PCB 87, PCB 99, PCB 101, PCB 105, PCB 114, PCB 118, PCB 119, PCB 123, PCB 126, PCB 128, PCB 138, PCB 138/158, PCB 146, PCB 151, PCB 153, PCB 156, PCB 157, PCB 164/163, PCB 167, PCB 169, PCB 170, PCB 177, PCB 178, PCB 180, PCB 182/187, PCB 183, PCB 187, PCB 189, PCB 194, PCB 196/203, PCB 199, PCB 201, PCB 206, PCB 209	Industrial use as electrical insulating and heat-exchange fluids [31]	Ingestion [32]	Increased risk of certain cancers, endocrine problems, and low birth weight [33]
PFAS	PFOA, PFOS, PFHxS, PFDA, PFNA, PFBS, PFHxA, PFHpA, PFUnDA, PFDoDA, PFPeA, PFTrDA, PFPeS, PFDS, FTS, PFHpS, PFNS	Used in industrial applications for non-stick or stain-resistant coatings, including in fabrics, paints, and finishes applied to common household products or packaging in food items [34, 35]	Ingestion [36]	Endocrine disruption and dysregulation of thyroid, immune, and reproductive systems [36]
Phthalates	MEOHP*, MEHHP*, MnBP*, MiBP*, MEHP*, MEP*, MECPP*, MCMHP*, MBzP*, DEHP, DnBP, DiBP, DcHP, DAP, DEP, DBP, BBzP, DOP, 7OH-MMeOP, DiNP, MiNP	Used to soften plastics in toys, personal care products, and food processing materials [37–39]	Ingestion, dermal absorption [37]	Negative impacts on neurological, endocrine, and reproductive development [38]. Early-life exposure is linked with adverse effects on mental and motor development [39, 40].

## Results

All chemical classes were detected in at least ten studies worldwide from 2004 to 2024. Results for each chemical class are listed below, with proposed mechanisms for maternal transfer of chemicals into human milk, countries in which chemicals were detected most frequently, most commonly detected compounds, and the maximum concentration detected out of all compounds in the chemical class.

### Bisphenols [12–15, 23, 41–46]

Despite being rapidly metabolized and excreted, research suggests repeated exposure to bisphenols through household items may lead to partial distribution to body compartments such as adipose tissue or human milk [42]. BPA is a lipophilic chemical, suggesting that it can enter human milk [12]. Upon passage into the mammary gland from the blood, bisphenols are excreted in human milk [47].

**Fig. 3:**
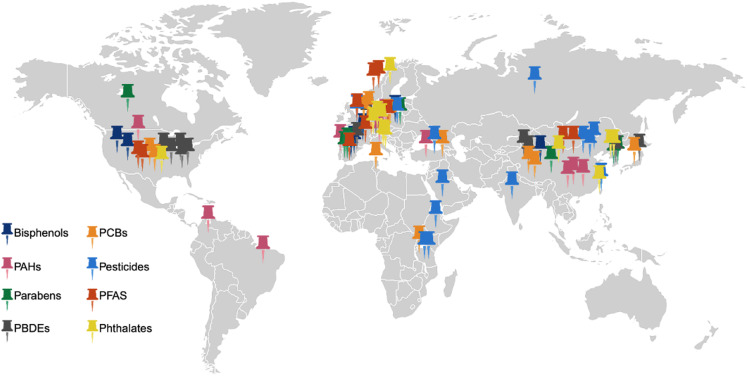
Location of EDC detection by chemical class, based on the country each study was published in from our review. Created in BioRender. Manz, K. (2025) https://www.biorender.com/

Bisphenols (BPA and analogs) have been detected in human milk in numerous countries, most frequently in South Korea, Spain, and the United States (Fig. 3). Of 11 studies (published from 2008 to 2022) measuring BPA concentrations in human milk, other bisphenol analogs were detected, including Bisphenol S (BPS) (3 studies), Bisphenol AF (BPAF) (2 studies), and Bisphenol F (BPF) (1 study). Of these chemicals, BPA was detected at the highest concentration at 29.9 ng/mL (Table S1**)**.

### Organochlorine Pesticides[16–18, 48–56]

As highly lipophilic chemicals, OCPs accumulate in maternal adipose tissue and are detectable bound to lipids in human milk [53]. Studies demonstrate that lactation lowers the maternal body burden of OCPS, leading to infant exposure to OCPs excreted in human milk [17, 55]. Health implications of pre- and post-natal exposure include endocrine disruption and increased risk for allergies, cancer, and metabolic, reproductive, and neurodevelopmental disorders [16].

In our review, 36 different OCPs were detected in human milk, with two studies each from Belgium and China and other studies distributed throughout Asia, Africa, and Europe. From the 11 studies identified (published from 2011 to 2022), the most commonly detected OCPs were p, p-DDT (10 studies) as well as its metabolites (p, p-DDE (ten studies) and p, p-DDD (seven studies), β-HCH (eight studies), and γ-HCH (six studies). From the chemicals detected, the maximum level of any OCP discovered in human milk was 28965.9 ng/g lipid of p, p- DDE. All results for OCPs detected can be found in Table S6.

### PAHs [19–22, 48, 49, 57–60]

As lipophilic chemicals, PAHs can be absorbed into the bloodstream and often bioaccumulate in adipose tissues [20]. Although these chemicals are rapidly excreted, chronic PAH intake, such as through smoking, increases the risk of accumulation in the body [22]. Such chronic exposure causes PAHs to accumulate in adipose tissue due to their lipophilic properties, which can then mobilize from adipose tissue into human milk during lactation [59].

PAH parent compounds are detected in human milk worldwide, most frequently in China, throughout Europe, and South America. Across these studies (published from 2008 to 2024), the most frequently detected chemicals included phenanthrene (10 studies), pyrene (10 studies), and fluoranthene (9 studies). Of all chemicals detected, naphthalene was recorded at the highest concentration at 1,477 ng/g lipid (Table S2).

### Parabens [15, 23, 24, 41–44, 61–63]

Most commonly, parabens are rapidly metabolized to p-hydroxybenzoic acid and excreted in urine [25]. However, in cases of chronic exposure, parabens that survive first-pass metabolism can bind to human serum albumin, protecting them from hydrolysis and enabling freeform paraben to bioaccumulate in other tissues [64]. Despite their rapid excretion in urine, continued exposure to high concentrations of parabens in personal care products causes parabens to be detectable in human milk, leading to possible postnatal paraben exposure for breastfeeding infants [65].

Parabens are detected in human milk worldwide. Of the ten studies reviewed (published from 2008 to 2023), seven parabens were identified. Of these seven parabens, the most frequently detected chemicals were methylparaben (10 studies), ethylparaben (nine studies), and propylparaben (nine studies). With regards to methylparaben, the maximum concentration detected in human milk was 40 ng/mL (Table S3).

### PBDEs [7, 11, 27–30, 50, 61, 66–69]

Due to their lipophilic nature, PDBEs accumulate in fatty tissues and are excreted during breastfeeding, causing a transfer of body burden of PBDEs from mother to child [29, 70].

Across 10 studies (published from 2006 to 2016), the most commonly detected PBDE chemicals in human milk were BDE-47, BDE-100, and BDE-153, all of which were detected in all 10 studies, with the most common countries in which PBDE has been studied being the USA (four studies) and China (two studies). From all PDBEs detected (18 chemicals), the maximum level detected was 1,430 ng/g lipid of BDE-47 (Table S4).

### PCBs [11, 16, 48–50, 61, 66–68, 71–73]

Since PCBs are highly lipophilic, they tend to concentrate in adipose tissues and are thus often excreted via lactation [74, 75].

PCBs are detected frequently in human milk throughout Europe, as well as throughout the United States and Asia. Of the ten studies reviewed (published from 2006 to 2019), 45 different PCB congeners were detected, with PCB118 and PCB153 being the most commonly identified as they were present in all ten studies reviewed. However, PCB180 was detected at the highest concentration, reaching levels up to 3,012 ng/g lipid (Table S5).

###  PFAS [34–36, 50, 76–83]

PFAS are structurally similar to free fatty acids; thus PFAS tend to bind proteins [80]. PFAS thus tends to be excreted bound to proteins in human milk, particularly in the early stages of lactation when protein levels are highest in human milk [80]. Reductions in maternal PFAS serum levels before and after lactation indicate that excretion of PFAS in human milk could be a concerning source of exposure to PFAS for nursing infants [84].

PFAS have been detected in human milk worldwide, with China, Norway, Spain, and the USA each having at least two independent studies and cohorts. Across 12 studies of PFAS in human milk (published from 2008 to 2024), the most commonly detected PFAS in human milk were PFOA (12 studies), PFOS (10 studies), and PFDA (seven studies). The levels at which each chemical was detected varied, with maxima reaching up to 1.1 ng/mL (PFOA), 0.87 ng/mL (PFOS), and 1.1 ng/mL (PFDA) (Table S7).

### Phthalates [15, 37–40, 61, 85–90]

After ingestion of phthalates, phthalate diesters are metabolized into monoester metabolites, leading to the detection of both unmetabolized diesters and their monoester metabolites in biological fluid samples (urine, serum, and human milk) [13, 47]. Upon exposure or ingestion, phthalates are excreted rapidly (within 24–48 h) [47]. However, phthalate exposure is often chronic due to the regular ingestion of contaminated food.

Phthalates are detected in human milk across many countries, mainly in East Asia and Europe (Fig. 3). Across 11 studies (published from 2008 to 2024), phthalates and their monoester metabolites were detected in both aqueous and lipid-linked samples, with units for measurement varying by study (eight studies utilized units of ng phthalates/mL, while three of the studies used a measurement of ng phthalates/g lipid, with one overlapping study using both measurements depending on the phthalate detected). We converted units of lipid-adjusted values (used in three studies) to fluid-adjusted values so that we could compare minimum and maximum values detected for each chemical (Supplemental Materials: Appendix A). Due to the metabolized nature of many phthalate chemicals detected, it is most effective to quantify the metabolized version of each chemical in human milk, as unmetabolized forms of phthalates may be a result of contamination during detection procedures. The three most commonly detected monoester metabolites were MEHP (eight studies), MiBP (seven studies), and MnBP (five studies) (Table S8).

## Discussion

### Factors Potentially Influencing Concentration Patterns

**Fig. 4:**
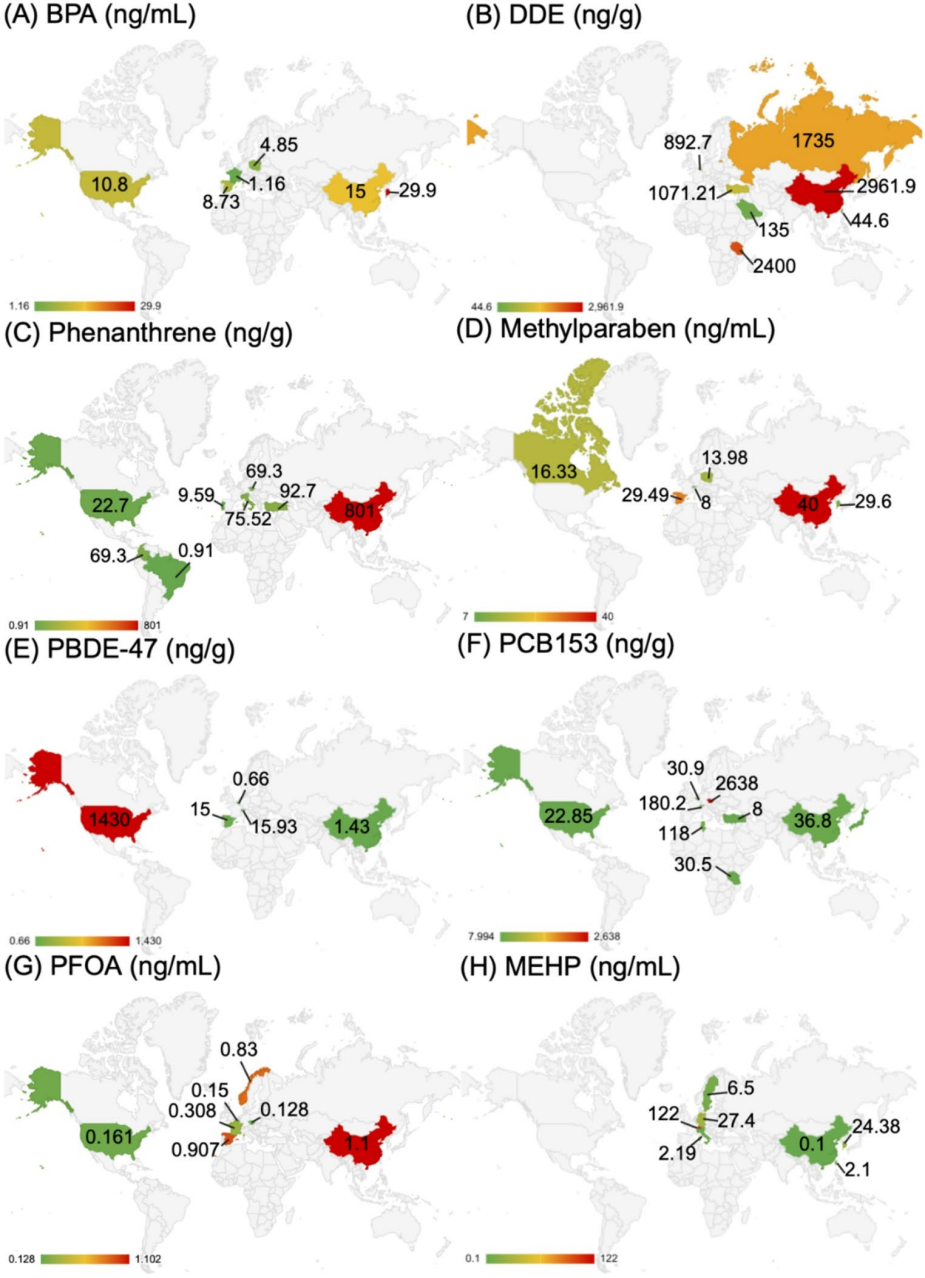
**A**) - **H**) depict the range and location of maximum concentrations detected for the most observed chemical of each class: (**A**) Bisphenols (BPA), (**B**) PAHs (phenanthrene), (**C**) parabens (methylparaben), (**D**) PBDEs (PBDE-47), (**E**) PCBs (PCB153), (**F**) pesticides (DDE), (**G**) PFAS (PFOA), (**H**) Phthalates (MEHP) https://www.biorender.com/

There are several factors which may influence the concentration of chemicals in human milk found in this review. Firstly, many chemicals studied in this review have varied in their use since 2004; government bans on the use of some EDCs in consumer products and for industrial purposes have altered the scope of chemical exposures over time [91]. The policies which ban the use of certain EDCs have not been enacted uniformly or at the same time [91], which may explain some of the geographic variation observed in Fig. 4. For example, the highest concentration of BDE-153 observed was 1,430 ng/g lipid in the United States (Fig. 4F), observed in samples collected from 2004 to 2006. However, the United States did not officially enact policies to regulate the production and use of PBDEs until 2006 [92], after the majority of samples in the study had been collected. Additionally, bans on chemical use have led to the development of replacement chemicals which have not yet been studied or regulated [91], thus potentially changing the concentrations of different chemicals found in human milk over time. In our analysis, we did not observe clear trends in chemical concentrations over time (Figure S1), indicating that further testing in specific geographic regions or of at-risk populations may help to identify children at risk of high EDC exposure through lactation.

**Fig. 5:**
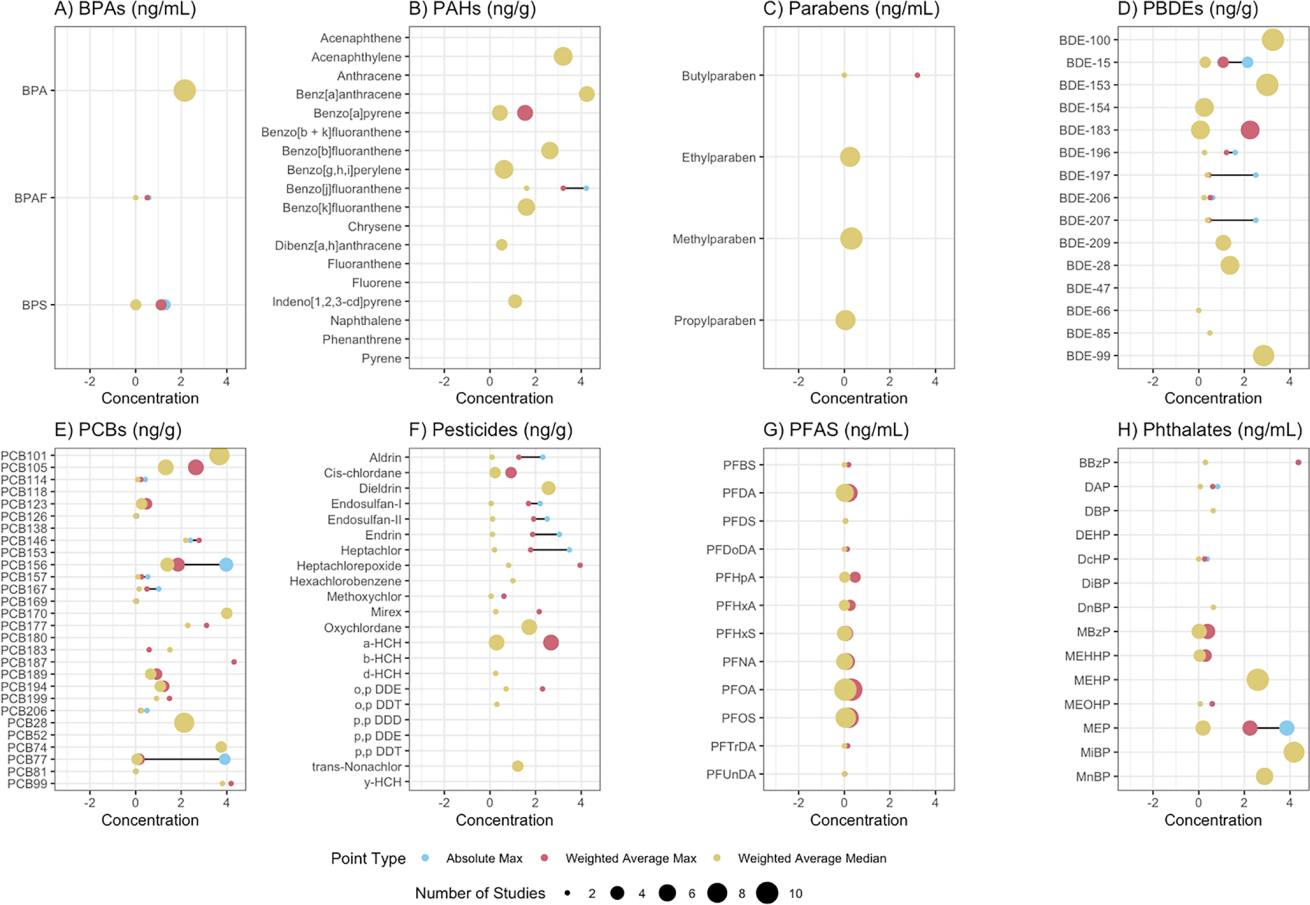
Concentration bubble plots for each compound found in more than one of the reviewed studies, arranged by chemical class (**A**) - **H**)). The three concentration values included for each chemical are: the absolute maximum value detected (blue), the weighted average of maxima for each chemical across all studies in which that chemical was detected (red), and the weighted average of the median for each chemical across all studies in which that chemical was detected (yellow)

Exposure to chemicals can also vary geographically or by population studied (Fig. 5), as the use of certain chemicals may vary by a country or region’s industrial profile or needs. For example, near a US Superfund Site contaminated with Dieldrin and DDT in Maryland, USA, every mile increase in residential distance from the site corresponded with a 1.6 ng/g lipid concentration decrease in blood Dieldrin levels in residents living near the site [93]. This study demonstrates at only a small scale (up to 28.33 miles from the site) how environmental exposures vary spatially. Further factors which may impact population-specific exposures include occupation, such as agricultural workers having higher exposure to pesticides due to occupational exposure. For example, a study of farmworkers vs. non-farmworkers found significantly higher levels of the pesticide 2,4-Dichlorophenoxyacetic Acid in farmworkers, suggesting that occupation may have an impact on individual EDC exposure [94].

Another factor that may contribute to the varying concentrations detected is differing milk collection times across studies. Studies in this review reported collection time of milk following pregnancy ranging from less than one week to nine months postpartum. Since this timing is not consistent across studies, it is more difficult to make accurate comparisons of these concentrations due to the changing composition of human milk throughout lactation. For example, colostrum (milk produced up to 2–5 days after birth) is high in protein and low in fat, whereas mature milk has a higher fat content and decreased protein content [95, 96]. Further, concentrations could be dependent on whether a full expression of milk was collected. Foremilk, which is produced at the beginning of a feed, can be thinner and have a watery composition; however, hindmilk, which flows towards the end of a feeding session, is higher in fat. These compositional differences in milk composition may impact the concentration of lipophilic compounds (like many of the EDCs studied in this review) found in milk in different phases of lactation.

In addition to the factors described above, laboratory and methodological considerations also play a critical role in interpreting chemical concentration data in human milk. Sample contamination during milk collection, storage, or laboratory processing can introduce artifacts that may impact reported concentrations. To mitigate these issues, it is essential to standardize collection procedures, thoroughly document the type of pump and materials used during collection, and incorporate the use of field blanks, procedural blanks, and quality control materials. The inclusion and thorough reporting of such quality assurance/quality control measures in future studies will be important for ensuring valid cross-study comparisons and accurate exposure assessment.

### Health Implication Patterns

Several studies noted health implications (either detected in the study or inferred from other literature) associated with the recorded concentrations of respective chemicals. Of the 71 studies analyzed in this review, the most prominent health impacts associated with early-life EDC exposure in human milk reported included neurodevelopmental toxicity and alteration or disruption of thyroid hormone levels.

With regards to neurodevelopmental toxicity, adverse effects identified in this review were associated with increased PBDE and OCP concentrations. Gascon et al. tested for this association with regards to colostrum PBDE concentrations and discovered higher PBDE exposure across all analogs detected are associated with lower Bayley Scales of Infant and Toddler Development scores (with higher scores indicating better development) [28]. A similar trend was observed by Kao et al., testing and discovering that numerous OCPs in human milk were inversely associated with Bayley cognitive and language outcomes later on in the infant’s life [56]. An additional systematic review conducted by Ramírez et al. found associations between early postnatal exposure to EDCs and neurodevelopmental and behavioral abnormalities/disorders in chemical classes including PBDEs, pesticides, bisphenols, phthalates, PCBs, and PFAS [97]. For example, higher levels of pesticides including β-hexachlorocyclohexane and 2,4,6-trichloropropane or metabolites of pesticides such as dimethyl phosphate and dimethyl alkyl phosphate were associated with greater risk of ADHD [97]. Additionally, they found that children whose mothers presented higher levels of PBDE-47 in breast milk were 3.3 times more likely to have increased externalizing behaviors, such as impulsivity, according to the ITSEA (Infant-Toddler Social and Emotional Assessment) [97].

In addition to neurodevelopmental toxicity, numerous studies found associations between EDC concentrations and altered thyroid hormone levels. One study noted a correlation between thyroid hormone imbalance and PBDE accumulation, specifically in human milk shortly following delivery [7]. Similarly, another study found an inverse association between some OCP concentrations (list which OCPs) in human milk and thyroid stimulating hormone (TSH) and IGF-1 levels [56]. Egalini et al. concluded in their review that exposure to PCBs, PBDEs, phthalates, bisphenols, and certain pesticides, all have the potential to interfere with thyroid hormone receptors across the lifespan [98].

## Risk Assessment of Exposure

Several studies compared daily intake of EDCs in human milk to existing reference doses (RfDs) or tolerable daily intake values (TDIs) (based on daily ingestion of a specific chemical or chemical class) at the time of the study (which vary by the country and year in which the study was conducted and the chemical or chemical class of interest). Based on levels of EDCs found in human milk, 13 studies found that infants were ingesting higher levels of EDCs than recommended through human milk (Table S9). All chemical classes included in this review were discovered in at least one study at a level higher than recommended RfD or TDI. However, even among the measures used in these studies, there were inconsistencies across the analysis of daily intake doses; only two out of 13 studies used an additional 10-fold margin for risk recommended for infants, while others assessed neonatal exposure using the same values as adults (adjusted for infant weight). In future studies, a standard measure accounting for the vulnerability of infants for early-life exposure to EDCs (such as the additional 10-fold margin used by two of the studies) should be used in assessing the risk to infants of ingestion of EDCs through human milk. The United States Environmental Protection Agency suggests investigating the toxicity risks and adjusting the risk inflation factor for infants (advised to be 10-fold currently) associated with individual chemicals on a case-by-case basis, as the mechanism of bioaction may vary by chemical in humans [99]. Additionally, health guidance values specific to concentrations of EDCs in human milk would provide particularly useful information for breastfed infants, rather than extrapolations from measures specific to adult ingestion or water concentrations.

Of note, Jin et al., Czarczyńska-Goślińska et al., Galindo et al., Wang et al., Yu et al., Çok et al., Kim et al., Park et al., Bramwell et al., Jin et al., Tao et al., Liu et al., Fromme et al., and Guerranti et al. assessed health risks (with either RfDs or TDIs) and found that levels of EDCs in human milk were not above recommended levels, indicating that there may be geographic or temporal variability in the excess risk posed to nursing infants by maternal lifetime exposure and EDC body burden at the time of lactation [12, 23, 24, 37, 39, 40, 48, 57–59, 69, 76, 77, 85].

## Trends in Compound Depuration

Five studies identified varying longitudinal trends of PBDEs, PCBs, pesticides, PFAS, and phthalates in human milk (Table S10). Some studies contradicted each other in the trends noted for the same chemicals; for example, two studies observed opposing trends for PFAS concentrations in human milk throughout lactation, with one concluding an increase in PFAS concentration in human milk from 0.5 to three months postpartum and another finding a decrease over a one-year lactation period. The differences in EDC concentrations over the lactation period may demonstrate variation in the mobilization of different chemicals, indicating that nursing infants may be at risk of exposure to a mixture of chemicals in human milk, which vary in concentration over time. Further longitudinal studies over controlled time periods while taking potential exposures and location into account are needed to understand the trends in concentrations of EDCs in human milk as well as the potential risks associated with exposure to mixtures of EDCs in neonates.

### Notes for Future Studies

In addition to the need for a greater number of studies on the geographic and longitudinal distribution of EDCs in human milk, it is important to assess the composition of human milk, EDCs in human milk, and appropriate laboratory protocols (such as freeze/thaw cycles and addressing/quantifying any potential contamination of samples from the collection materials) that may impact the quality of results obtained. Several factors need to be considered in each study’s results, including the potential for environmental factors to increase chemical exposures, and the possibility of contamination of samples during collection and analysis. For example, the use of plastics during milk collection could increase levels of bisphenols in sample results unless proper control samples are included; in addition, proper upfront method development is required to ensure that milk does not leach bisphenols from collection materials [100]. Further standardization of human milk collection and analysis techniques are also needed to ensure comparable results across studies, as utilizing complementary measurement techniques (liquid or gas chromatography) and universal methods of collection and analysis could enhance coverage and concentrations of the chemicals detected [101].

Lastly, to truly understand the risks posed to nursing infants, it is essential to understand the transfer of chemicals into human milk as well as how maternal body burden relates to EDC levels in human milk. Using matched serum, urine, and human milk samples over a defined period to detect multiple classes of EDCs would provide the most comprehensive picture of EDC exposure in neonates due to nursing. Additionally, there is a need for a greater number of studies including diverse populations to determine the full range and variability of neonatal EDC exposure and to ensure that findings are more broadly applicable to different racial and socioeconomic groups. Finally, it is essential to contextualize the concentrations and trends detected with either the current use or lack thereof of specific compounds, as compounds which have been phased out of use may be present at lower levels in comparison to previous studies or studies located where such compounds are still in use.

## Conclusion

As human milk is the main and often sole source of nutrition for many infants, it is essential to understand the extent to which nursing infants are exposed to EDCs and quantify any associated health risks. In this review, we examined 70 published studies with human milk samples collected during or after 2004 that provided concentration ranges for EDCs. Across the eight chemical classes we focused on, the highest chemical concentrations detected were observed in the OCP and PCB categories. However, there is a need for continued research across all classes of EDCs to comprehensively characterize EDC exposure in infants and understand the relationship between maternal body burden and human milk EDC levels.

In future studies, there should be a focus on improving detection techniques, incorporating quality control measures, and assessing EDC exposure across multiple biological matrices over time to obtain more precise exposure estimates in nursing infants. Further, more robust data is needed to characterize EDC levels by both population and region and to clarify their associations with adverse health outcomes to inform more comprehensive lactation recommendations.

## Key References

- Czosnykowska-Łukacka M, Królak-Olejnik B, Orczyk-Pawiłowicz M. Breast Milk Macronutrient Components in Prolonged Lactation. Nutrients. 2018;10.
  - ○ The study outlines the immediate and long-term benefits of breastfeeding and establishes breastfeeding as the “gold standard” for early life nutrition.
- Schlumpf M, Kypke K, Wittassek M, Angerer J, Mascher H, Mascher D, et al. Exposure patterns of UV filters, fragrances, parabens, phthalates, organochlor pesticides, PBDEs, and PCBs in human milk: Correlation of UV filters with use of cosmetics. Chemosphere. 2010;81:1171–83.
  - ○ The study determined that there were multiple classes of EDCs above the RfD in human milk; combinations of EDCs detected in human milk may be of particular concern for health impacts.
- Kao C-C, Que DE, Bongo SJ, Tayo LL, Lin Y-H, Lin C-W, et al. Residue Levels of Organochlorine Pesticides in Breast Milk and Its Associations with Cord Blood Thyroid Hormones and the Offspring’s Neurodevelopment. Int J Environ Res Public Health. 2019;16:1438
  - ○ The study identified neurological and endocrine disruptions as a result of early-life exposure to OCPs.
- Zhang D, Xiao J, Xiao Q, Chen Y, Li X, Zheng Q, et al. Infant exposure to parabens, triclosan, and triclocarban via breastfeeding and formula supplementing in southern China. Sci Total Environ. 2023;858:159820.
  - ○ The study used a 10-fold margin of safety for infants to determine whether or not exposure to EDCs were at a concerning level. This safety margin demonstrates that comprehensive research on safety levels specified by age would help advise future public health guidelines.
- LaKind Judy S., Berlin Cheston M., Sjödin Andreas, Turner Wayman, Wang Richard Y., Needham Larry L., et al. Do Human Milk Concentrations of Persistent Organic Chemicals Really Decline During Lactation? Chemical Concentrations During Lactation and Milk/Serum Partitioning. Environ Health Perspect. 2009;117:1625–31.
  - ○ This study demonstrated no decline in the concentrations of PBDEs, OCPs, and PFAS over the lactation period. This information differs from other results, indicating the need for comprehensive studies with many participants over a specified time period to determine EDC concentration changes throughout lactation.

## Supplementary Information

Below is the link to the electronic supplementary material.

Supplementary Material 1 (PDF 356 KB)

Supplementary Material 2 (PDF 807 KB)

## Data Availability

No datasets were generated or analysed during the current study.

## References

[1] Recommendations. and Benefits | Nutrition | CDC [Internet]. [cited 2024 Nov 19]. Available from: https://www.cdc.gov/nutrition/infantandtoddlernutrition/breastfeeding/recommendations-benefits.html

[2] Breastfeeding. [Internet]. [cited 2024 Nov 19]. Available from: https://www.who.int/health-topics/breastfeeding

[3] Brahm P, Valdes V. Benefits of breastfeeding and risks associated with not breastfeeding. Rev Chil Pediatr. 2017;88:15–21. 10.4067/S0370-41062017000100001 28288222

[4] Thai JD, Gregory KE. Bioactive factors in human breast milk attenuate intestinal inflammation during early life. Nutrients. 2020. 10.3390/nu12020581. 32102231 10.3390/nu12020581 PMC7071406

[5] Czosnykowska-Łukacka M, Królak-Olejnik B, Orczyk-Pawiłowicz M. Breast milk macronutrient components in prolonged lactation. Nutrients. 2018. 10.3390/nu10121893. 30513944 10.3390/nu10121893 PMC6316538

[6] Kabir ER, Rahman MS, Rahman I. A review on endocrine disruptors and their possible impacts on human health. Environ Toxicol Pharmacol. 2015;40:241–58. 26164742 10.1016/j.etap.2015.06.009

[7] Kim U-J, Lee I-S, Kim HS, Oh J-E. Monitoring of PBDEs concentration in umbilical cord blood and breast milk from Korean population and estimating the effects of various parameters on accumulation in humans. Chemosphere. 2011;85:487–93. 21890170 10.1016/j.chemosphere.2011.08.008

[8] Zoeller RT, Bergman Å, Becher G, Bjerregaard P, Bornman R, Brandt I, et al. A path forward in the debate over health impacts of endocrine disrupting chemicals. Environ Health. 2014;13:118. 25533907 10.1186/1476-069X-13-118 PMC4298083

[9] Kahn LG, Philippat C, Nakayama SF, Slama R, Trasande L. Endocrine-disrupting chemicals: implications for human health. Lancet Diabetes Endocrinol. 2020;8:703–18. 32707118 10.1016/S2213-8587(20)30129-7 PMC7437820

[10] Li A, Zhuang T, Song M, Cao H, Gao Y, Zheng S, et al. Occurrence, placental transfer, and health risks of emerging endocrine-disrupting chemicals in pregnant women. J Hazard Mater. 2023;459:132157. 37506642 10.1016/j.jhazmat.2023.132157

[11] LaKind JS, Berlin Cheston M, Sjödin Andreas T, Wayman WRY, Needham Larry L, et al. Do human milk concentrations of persistent organic chemicals really decline during lactation? Chemical concentrations during lactation and milk/serum partitioning. Environ Health Perspect. 2009;117:1625–31. 20019916 10.1289/ehp.0900876 PMC2790520

[12] Jin H, Xie J, Mao L, Zhao M, Bai X, Wen J, et al. Bisphenol analogue concentrations in human breast milk and their associations with postnatal infant growth. Environ Pollut. 2020;259:113779. 31887597 10.1016/j.envpol.2019.113779

[13] Yi B, Kim C, Yang M. Biological monitoring of bisphenol A with HLPC/FLD and LC/MS/MS assays. Biol Monit Anal Toxicol Occup Environ Med. 2010;878:2606–10. 10.1016/j.jchromb.2010.02.008 20202916

[14] Zimmers SM, Browne EP, O’Keefe PW, Anderton DL, Kramer L, Reckhow DA, et al. Determination of free bisphenol A (BPA) concentrations in breast milk of U.S. women using a sensitive LC/MS/MS method. Chemosphere. 2014;104:237–43. 24507723 10.1016/j.chemosphere.2013.12.085

[15] Kim JH, Kim D, Moon S-M, Yang EJ. Associations of lifestyle factors with phthalate metabolites, bisphenol A, parabens, and triclosan concentrations in breast milk of Korean mothers. Chemosphere. 2020;249:126149. 32062213 10.1016/j.chemosphere.2020.126149

[16] Müller MHB, Polder A, Brynildsrud OB, Karimi M, Lie E, Manyilizu WB, et al. Organochlorine pesticides (OCPs) and polychlorinated biphenyls (PCBs) in human breast milk and associated health risks to nursing infants in Northern Tanzania. Environ Res. 2017;154:425–34. 28196346 10.1016/j.envres.2017.01.031

[17] Polder A, Gabrielsen GW, Odland JØ, Savinova TN, Tkachev✠ A, Løken KB, et al. Spatial and temporal changes of chlorinated pesticides, PCBs, dioxins (PCDDs/PCDFs) and brominated flame retardants in human breast milk from Northern Russia. Sci Total Environ. 2008;391:41–54. 18063018 10.1016/j.scitotenv.2007.10.045

[18] Witczak A, Pohoryło A, Abdel-Gawad H. Endocrine-disrupting organochlorine pesticides in human breast milk: changes during lactation. Nutrients. 2021. 10.3390/nu13010229. 33466783 10.3390/nu13010229 PMC7830316

[19] Santonicola S, De Felice A, Cobellis L, Passariello N, Peluso A, Murru N, et al. Comparative study on the occurrence of polycyclic aromatic hydrocarbons in breast milk and infant formula and risk assessment. Chemosphere. 2017;175:383–90. 28236708 10.1016/j.chemosphere.2017.02.084

[20] Oliveira M, Duarte S, Delerue-Matos C, Pena A, Morais S. Exposure of nursing mothers to polycyclic aromatic hydrocarbons: levels of un-metabolized and metabolized compounds in breast milk, major sources of exposure and infants’ health risks. Environ Pollut. 2020;266:115243. 32702605 10.1016/j.envpol.2020.115243

[21] Torres-Moreno C, Puente-DelaCruz L, Codling G, Villa AL, Cobo M, Klanova J, et al. Polycyclic aromatic hydrocarbons (PAHs) in human breast milk from Colombia: spatial occurrence, sources and probabilistic risk assessment. Environ Res. 2022;204:111981. 34499895 10.1016/j.envres.2021.111981

[22] Pulkrabova J, Stupak M, Svarcova A, Rossner P, Rossnerova A, Ambroz A, et al. Relationship between atmospheric pollution in the residential area and concentrations of polycyclic aromatic hydrocarbons (PAHs) in human breast milk. Sci Total Environ. 2016;562:640–7. 27107652 10.1016/j.scitotenv.2016.04.013

[23] Czarczyńska-Goślińska B, Grześkowiak T, Frankowski R, Lulek J, Pieczak J, Zgoła-Grześkowiak A. Determination of bisphenols and parabens in breast milk and dietary risk assessment for Polish breastfed infants. J Food Compos Anal. 2021;98:103839.

[24] Park N-Y, Cho YH, Choi K, Lee E-H, Kim YJ, Kim JH, et al. Parabens in breast milk and possible sources of exposure among lactating women in Korea. Environ Pollut. 2019;255:113142. 31563777 10.1016/j.envpol.2019.113142

[25] Fransway AF, Fransway PJ, Belsito DV, Yiannias JA. Paraben toxicology. Dermatitis. 2019;30:32–45. 30570577 10.1097/DER.0000000000000428

[26] Chen M-H, Yu B, Zhang Z-F, Ma W-L. Occurrence of parabens in outdoor environments: implications for human exposure assessment. Environ Pollut. 2021;282:117058. 33838443 10.1016/j.envpol.2021.117058

[27] Sudaryanto A, Kajiwara N, Tsydenova OV, Isobe T, Yu H, Takahashi S, et al. Levels and congener specific profiles of PBDEs in human breast milk from China: implication on exposure sources and pathways. Chemosphere. 2008;73:1661–8. 18834613 10.1016/j.chemosphere.2008.07.088

[28] Mireia G, Joan FMMartínezDCA-EF, Grimalt Joan O, et al. Polybrominated diphenyl ethers (PBDEs) in breast milk and neuropsychological development in infants. Environ Health Perspect. 2012;120:1760–5. 23052368 10.1289/ehp.1205266 PMC3548276

[29] Daniels Julie L, Pan I-J, Richard J, Sarah A, Patterson Donald G, Needham Larry L, et al. Individual characteristics associated with PBDE levels in U.S. Human milk samples. Environ Health Perspect. 2010;118:155–60. 20056574 10.1289/ehp.0900759 PMC2831961

[30] Guo W, Holden A, Smith SC, Gephart R, Petreas M, Park J-S. Pbde levels in breast milk are decreasing in California. Chemosphere. 2016;150:505–13. 26693645 10.1016/j.chemosphere.2015.11.032

[31] Ross G. The public health implications of polychlorinated biphenyls (PCBs) in the environment. Ecotoxicol Environ Saf. 2004;59:275–91. 15388267 10.1016/j.ecoenv.2004.06.003

[32] Carpenter David O. Polychlorinated biphenyls (PCBs): routes of exposure and effects on human health. Rev Environ Health. 2006;21:1–24. 16700427 10.1515/reveh.2006.21.1.1

[33] Kimbrough RD, Krouskas CA. Human exposure to polychlorinated biphenyls and health effects. Toxicol Rev. 2003;22:217–33. 15189045 10.2165/00139709-200322040-00004

[34] Zhang X, Zhou X, Chen H, Gao X, Zhou Y, Lee HK, et al. Changes in concentrations of polyfluoroalkyl substances in human milk over lactation time and effects of maternal exposure via analysis of matched samples. Environ Sci Technol. 2024;58:4115–26. 38390687 10.1021/acs.est.3c09896

[35] Haug LS, Huber S, Becher G, Thomsen C. Characterisation of human exposure pathways to perfluorinated compounds — comparing exposure estimates with biomarkers of exposure. Environ Int. 2011;37:687–93. 21334069 10.1016/j.envint.2011.01.011

[36] Serrano L, Iribarne-Durán LM, Suárez B, Artacho-Cordón F, Vela-Soria F, Peña-Caballero M, et al. Concentrations of perfluoroalkyl substances in donor breast milk in Southern Spain and their potential determinants. Int J Hyg Environ Health. 2021;236:113796. 34192647 10.1016/j.ijheh.2021.113796

[37] Guerranti C, Sbordoni I, Fanello EL, Borghini F, Corsi I, Focardi SE. Levels of phthalates in human milk samples from central Italy. XIV Hung - Ital Symp Spectrochem Anal Tech Preserv Nat Resour Sumeg Hung Oct 5–7 2011. 2013;107:178–81.

[38] Hung S-C, Lin T-I, Suen J-L, Liu H-K, Wu P-L, Wu C-Y, et al. Phthalate exposure pattern in breast milk within a six-month postpartum time in southern Taiwan. Int J Environ Res Public Health. 2021. 10.3390/ijerph18115726. 34073581 10.3390/ijerph18115726 PMC8198263

[39] Fromme H, Gruber L, Seckin E, Raab U, Zimmermann S, Kiranoglu M, et al. Phthalates and their metabolites in breast milk — results from the Bavarian Monitoring of Breast Milk (BAMBI). Environ Int. 2011;37:715–22. 21406311 10.1016/j.envint.2011.02.008

[40] Kim S, Lee J, Park J, Kim H-J, Cho G, Kim G-H, et al. Concentrations of phthalate metabolites in breast milk in Korea: estimating exposure to phthalates and potential risks among breast-fed infants. Sci Total Environ. 2015;508:13–9. 25437948 10.1016/j.scitotenv.2014.11.019

[41] Ye X, Bishop AM, Needham LL, Calafat AM. Automated on-line column-switching HPLC-MS/MS method with peak focusing for measuring parabens, triclosan, and other environmental phenols in human milk. Anal Chim Acta. 2008;622:150–6. 18602546 10.1016/j.aca.2008.05.068

[42] Iribarne-Durán LM, Peinado FM, Freire C, Castillero-Rosales I, Artacho-Cordón F, Olea N. Concentrations of bisphenols, parabens, and benzophenones in human breast milk: a systematic review and meta-analysis. Sci Total Environ. 2022;806:150437. 34583069 10.1016/j.scitotenv.2021.150437

[43] Dualde P, Pardo O, Fernández F, Pastor S, Yusà A. Determination of four parabens and bisphenols A, F and S in human breast milk using quechers and liquid chromatography coupled to mass spectrometry. J Chromatogr B. 2019;1114–1115:154–66. 10.1016/j.jchromb.2019.03.004 30890302

[44] Azzouz A, Rascón AJ, Ballesteros E. Simultaneous determination of parabens, alkylphenols, phenylphenols, bisphenol A and triclosan in human urine, blood and breast milk by continuous solid-phase extraction and gas chromatography–mass spectrometry. J Pharm Biomed Anal. 2016;119:16–26. 26637951 10.1016/j.jpba.2015.11.024

[45] Mendonca K, Hauser R, Calafat AM, Arbuckle TE, Duty SM. Bisphenol A concentrations in maternal breast milk and infant urine. Int Arch Occup Environ Health. 2014;87:13–20. 23212895 10.1007/s00420-012-0834-9 PMC4381877

[46] Deceuninck Y, Bichon E, Marchand P, Boquien C-Y, Legrand A, Boscher C, et al. Determination of bisphenol A and related substitutes/analogues in human breast milk using gas chromatography-tandem mass spectrometry. Anal Bioanal Chem. 2015;407:2485–97. 25627788 10.1007/s00216-015-8469-9

[47] Gao Q, Niu Y, Wang B, Liu J, Zhao Y, Zhang J, et al. Estimation of lactating mothers’ daily intakes of bisphenol A using breast milk. Environ Pollut. 2021;286:117545. 34438484 10.1016/j.envpol.2021.117545

[48] Çok I, Mazmanci B, Mazmanci MA, Turgut C, Henkelmann B, Schramm K-W. Analysis of human milk to assess exposure to PAHs, PCBs and organochlorine pesticides in the vicinity mediterranean City Mersin, Turkey. Environ Int. 2012;40:63–9. 22280929 10.1016/j.envint.2011.11.012

[49] Tsang HL, Wu S, Leung CKM, Tao S, Wong MH. Body burden of pops of Hong Kong residents, based on human milk, maternal and cord serum. Environ Int. 2011;37:142–51. 20828823 10.1016/j.envint.2010.08.010

[50] Croes K, Colles A, Koppen G, Govarts E, Bruckers L, Van de Mieroop E, et al. Persistent organic pollutants (POPs) in human milk: a biomonitoring study in rural areas of Flanders (Belgium). Chemosphere. 2012;89:988–94. 22840535 10.1016/j.chemosphere.2012.06.058

[51] Mekonen S, Ambelu A, Wondafrash M, Kolsteren P, Spanoghe P. Exposure of infants to organochlorine pesticides from breast milk consumption in Southwestern Ethiopia. Sci Rep. 2021;11:22053. 34764390 10.1038/s41598-021-01656-x PMC8585979

[52] Dong Y, Yin S, Zhang J, Guo F, Aamir M, Liu S, et al. Exposure patterns, chemical structural signatures, and health risks of pesticides in breast milk: a multicenter study in China. Sci Total Environ. 2022;830:154617. 35307419 10.1016/j.scitotenv.2022.154617

[53] EL-Saeid MH, Hassanin AS, Bazeyad AY. Levels of pesticide residues in breast milk and the associated risk assessment. Saudi J Biol Sci. 2021;28:3741–4. 34220226 10.1016/j.sjbs.2021.04.062 PMC8241607

[54] Zhou J, Zeng X, Zheng K, Zhu X, Ma L, Xu Q, et al. Musks and organochlorine pesticides in breast milk from Shanghai, China: levels, temporal trends and exposure assessment. Ecotoxicol Environ Saf. 2012;84:325–33. 22921253 10.1016/j.ecoenv.2012.08.011

[55] Bedi JS, Gill JPS, Aulakh RS, Kaur P, Sharma A, Pooni PA. Pesticide residues in human breast milk: risk assessment for infants from Punjab, India. Sci Total Environ. 2013;463–464:720–6. 23850662 10.1016/j.scitotenv.2013.06.066

[56] Kao C-C, Que DE, Bongo SJ, Tayo LL, Lin Y-H, Lin C-W, et al. Residue levels of organochlorine pesticides in breast milk and its associations with cord blood thyroid hormones and the offspring’s neurodevelopment. Int J Environ Res Public Health. 2019;16:1438. 31018505 10.3390/ijerph16081438 PMC6517872

[57] Galindo MV, Hantao LW, Sampaio NMFM, Pessoto MA, Oliveira WdaS, Godoy HT. Analysis of polycyclic aromatic hydrocarbons in Brazilian human milk: a simple and effective approach. Food Control. 2025;167:110796.

[58] Yu Y, Wang X, Wang B, Tao S, Liu W, Wang X, et al. Polycyclic aromatic hydrocarbon residues in human milk, placenta, and umbilical cord blood in Beijing, China. Environ Sci Technol. 2011;45:10235–42. 22032748 10.1021/es202827g

[59] Wang L, Liu A, Zhao Y, Mu X, Huang T, Gao H, et al. The levels of polycyclic aromatic hydrocarbons (PAHs) in human milk and exposure risk to breastfed infants in petrochemical industrialized Lanzhou Valley, Northwest China. Environ Sci Pollut Res. 2018;25:16754–66. 10.1007/s11356-018-1799-3 29611127

[60] Kim SR, Halden RU, Buckley TJ. Polycyclic aromatic hydrocarbons in human milk of nonsmoking U.S. women. Environ Sci Technol. 2008;42:2663–7. 18505013 10.1021/es702275x

[61] Schlumpf M, Kypke K, Wittassek M, Angerer J, Mascher H, Mascher D, et al. Exposure patterns of UV filters, fragrances, parabens, phthalates, organochlor pesticides, PBDEs, and PCBs in human milk: correlation of UV filters with use of cosmetics. Chemosphere. 2010;81:1171–83. 21030064 10.1016/j.chemosphere.2010.09.079

[62] Zhang D, Xiao J, Xiao Q, Chen Y, Li X, Zheng Q, et al. Infant exposure to parabens, triclosan, and triclocarban via breastfeeding and formula supplementing in southern China. Sci Total Environ. 2023;858:159820. 36349623 10.1016/j.scitotenv.2022.159820

[63] Fisher M, MacPherson S, Braun JM, Hauser R, Walker M, Feeley M, et al. Paraben concentrations in maternal urine and breast milk and its association with personal care product use. Environ Sci Technol. 2017;51:4009–17. 28318231 10.1021/acs.est.6b04302

[64] Hager E, Chen J, Zhao L. Minireview: parabens exposure and breast cancer. Int J Environ Res Public Health. 2022;19:1873. 35162895 10.3390/ijerph19031873 PMC8834979

[65] Grecco CF, Souza ID, Queiroz MEC. Recent development of chromatographic methods to determine Parabens in breast milk samples: A review. J Chromatogr B. 2018;1093–1094:82–90. 10.1016/j.jchromb.2018.06.059 29990718

[66] Kim H, Jianwen S, Margaret S, Joan C, Nicholas J, Rosanne G, et al. Depuration of polybrominated Diphenyl ethers (PBDEs) and polychlorinated biphenyls (PCBs) in breast milk from California first-time mothers (Primiparae). Environ Health Perspect. 2007;115:1271–5. 17805415 10.1289/ehp.10166 PMC1964891

[67] Kayoko I, Kouji H, Katsunobu T, Shigeki U, Makoto K, Takashi S, et al. Levels and concentration ratios of polychlorinated biphenyls and polybrominated Diphenyl ethers in serum and breast milk in Japanese mothers. Environ Health Perspect. 2006;114:1179–85. 16882522 10.1289/ehp.9032 PMC1552037

[68] Shen H, Ding G, Wu Y, Pan G, Zhou X, Han J, et al. Polychlorinated dibenzo-p-dioxins/furans (PCDD/Fs), polychlorinated biphenyls (PCBs), and polybrominated diphenyl ethers (PBDEs) in breast milk from Zhejiang, China. Environ Int. 2012;42:84–90. 21575990 10.1016/j.envint.2011.04.004

[69] Bramwell L, Fernandes A, Rose M, Harrad S, Pless-Mulloli T. PBDEs and PBBs in human serum and breast milk from cohabiting UK couples. Flame Retard Environ - Pap Present 6th Int Symp Flame Retard BFR2013 San Franc April 7–10. 2014;116:67–74. 10.1016/j.chemosphere.2014.03.060 24745556

[70] Carrizo D, Grimalt JO, Ribas-Fito N, Sunyer J, Torrent M. Influence of breastfeeding in the accumulation of polybromodiphenyl ethers during the first years of child growth. Environ Sci Technol. 2007;41:4907–12. 17711201 10.1021/es070217u

[71] Soechitram SD, Chan SM, Nelson EAS, Brouwer A, Sauer PJJ. Comparison of dioxin and PCB concentrations in human breast milk samples from Hong Kong and the Netherlands. Food Addit Contam. 2003;20:65–9. 12519720 10.1080/0265203021000031528

[72] Ennaceur S, Gandoura N, Driss MR. Distribution of polychlorinated biphenyls and organochlorine pesticides in human breast milk from various locations in Tunisia: levels of contamination, influencing factors, and infant risk assessment. Environ Res. 2008;108:86–93. 18614165 10.1016/j.envres.2008.05.005

[73] Komprda J, Komprdová K, Domínguez-Romero E, Mikeš O, Řiháčková K, Čupr P, et al. Dynamics of PCB exposure in the past 50 years and recent high concentrations in human breast milk: analysis of influencing factors using a physiologically based pharmacokinetic model. Sci Total Environ. 2019;690:388–99. 31299572 10.1016/j.scitotenv.2019.06.504

[74] Rogan WJ, Gladen BC, McKinney JD, Carreras N, Hardy P, Thullen J, et al. Polychlorinated biphenyls (PCBs) and dichlorodiphenyl dichloroethene (DDE) in human milk: effects of maternal factors and previous lactation. Am J Public Health. 1986;76:172–7. 3080910 10.2105/ajph.76.2.172 PMC1646471

[75] Polychlorinated. Biphenyls, Nutrition, and Diabetes.

[76] Tao L, Kannan K, Wong CM, Arcaro KF, Butenhoff JL. Perfluorinated compounds in human milk from Massachusetts, U.S.A. Environ Sci Technol. 2008;42:3096–101. 18497172 10.1021/es702789k

[77] Jin H, Mao L, Xie J, Zhao M, Bai X, Wen J, et al. Poly- and perfluoroalkyl substance concentrations in human breast milk and their associations with postnatal infant growth. Sci Total Environ. 2020;713:136417. 31955077 10.1016/j.scitotenv.2019.136417

[78] Thomsen C, Haug LS, Stigum H, Frøshaug M, Broadwell SL, Becher G. Changes in concentrations of perfluorinated compounds, polybrominated diphenyl ethers, and polychlorinated biphenyls in Norwegian breast-milk during twelve months of lactation. Environ Sci Technol. 2010;44:9550–6. 21090747 10.1021/es1021922

[79] Criswell RL, Wang Y, Christensen B, Botelho JC, Calafat AM, Peterson LA, et al. Concentrations of per- and polyfluoroalkyl substances in paired maternal plasma and human milk in the new Hampshire birth cohort. Environ Sci Technol. 2022;57:463–72. 36574487 10.1021/acs.est.2c05555 PMC9837617

[80] Llorca M, Farré M, Picó Y, Teijón ML, Álvarez JG, Barceló D. Infant exposure of perfluorinated compounds: levels in breast milk and commercial baby food. Environ Int. 2010;36:584–92. 20494442 10.1016/j.envint.2010.04.016

[81] Zheng G, Schreder E, Dempsey JC, Uding N, Chu V, Andres G, et al. Per- and polyfluoroalkyl substances (PFAS) in breast milk: concerning trends for current-use PFAS. Environ Sci Technol. 2021;55:7510–20. 33982557 10.1021/acs.est.0c06978

[82] Cariou R, Veyrand B, Yamada A, Berrebi A, Zalko D, Durand S, et al. Perfluoroalkyl acid (PFAA) levels and profiles in breast milk, maternal and cord serum of French women and their newborns. Environ Int. 2015;84:71–81. 26232143 10.1016/j.envint.2015.07.014

[83] Lankova D, Lacina O, Pulkrabova J, Hajslova J. The determination of perfluoroalkyl substances, brominated flame retardants and their metabolites in human breast milk and infant formula. Talanta. 2013;117:318–25. 24209347 10.1016/j.talanta.2013.08.040

[84] Mondal Debapriya, Weldon Rosana Hernandez, Armstrong Ben G., Gibson Lorna J., Lopez-Espinosa Maria-Jose, Shin Hyeong-Moo, et al. Breastfeeding: a potential excretion route for mothers and implications for infant exposure to perfluoroalkyl acids. Environ Health Perspect. 2014;122:187–92. 24280536 10.1289/ehp.1306613 PMC3915259

[85] Liu Y, Xiao M, Huang K, Cui J, Liu H, Yu Y, et al. Phthalate metabolites in breast milk from mothers in Southern China: occurrence, temporal trends, daily intake, and risk assessment. J Hazard Mater. 2024;464:132895. 37976856 10.1016/j.jhazmat.2023.132895

[86] Hines EP, Calafat AM, Silva MJ, Mendola P, Fenton SE. Concentrations of phthalate metabolites in milk, urine, saliva, and serum of lactating North Carolina women. Environ Health Perspect. 2009;117:86–92. 19165392 10.1289/ehp.11610 PMC2627871

[87] Zimmermann S, Gruber L, Schlummer M, Smolic S, Fromme H. Determination of phthalic acid diesters in human milk at low Ppb levels. Food Addit Contam Part A. 2012;29:1780–90. 10.1080/19440049.2012.704529 22845555

[88] Latini G, Wittassek M, Del Vecchio A, Presta G, De Felice C, Angerer J. Lactational exposure to phthalates in Southern Italy. Environ Int. 2009;35:236–9. 18684505 10.1016/j.envint.2008.06.002

[89] Högberg J, Hanberg A, Berglund M, Skerfving S, Remberger M, Calafat AM, et al. Phthalate diesters and their metabolites in human breast milk, blood or serum, and urine as biomarkers of exposure in vulnerable populations. Environ Health Perspect. 2008;116:334–9. 18335100 10.1289/ehp.10788 PMC2265037

[90] Zhu J, Phillips SP, Feng Y-L, Yang X. Phthalate esters in human milk: concentration variations over a 6-month postpartum time. Environ Sci Technol. 2006;40:5276–81. 16999099 10.1021/es060356w

[91] Duh-Leong C, Maffini MV, Kassotis CD, Vandenberg LN, Trasande L. The regulation of endocrine-disrupting chemicals to minimize their impact on health. Nat Rev Endocrinol. 2023;19:600–14. 37553404 10.1038/s41574-023-00872-x

[92] Sutton R, Sedlak MD, Yee D, Davis JA, Crane D, Grace R, et al. Declines in polybrominated diphenyl ether contamination of San Francisco Bay following production phase-outs and bans. Environ Sci Technol. 2015;49:777–84. 25544014 10.1021/es503727b

[93] Gaffney Shannon H., Curriero Frank C., Strickland Paul T., Glass Gregory E., Helzlsouer Kathy J., Breysse Patrick N. Influence of geographic location in modeling blood pesticide levels in a community surrounding a U.S. Environmental Protection Agency Superfund Site. Environ Health Perspect. 2005;113:1712–6. 16330352 10.1289/ehp.8154 PMC1314910

[94] Forté CA, Millar JA, Colacino JA. Integrating NHANES and toxicity forecaster data to compare pesticide exposure and bioactivity by farmwork history and US citizenship. J Expo Sci Environ Epidemiol. 2024;34:208–16. 37474644 10.1038/s41370-023-00583-5 PMC10799167

[95] Breastfeeding. and Delayed Milk Production [Internet]. 2024 [cited 2025 Aug 1]. Available from: https://www.hopkinsmedicine.org/health/conditions-and-diseases/breastfeeding-and-delayed-milk-production

[96] Andreas NJ, Kampmann B, Mehring Le-Doare K. Human breast milk: a review on its composition and bioactivity. Early Hum Dev. 2015;91:629–35. 26375355 10.1016/j.earlhumdev.2015.08.013

[97] Ramírez V, Gálvez-Ontiveros Y, González-Domenech PJ, Baca MÁ, Rodrigo L, Rivas A. Role of endocrine disrupting chemicals in children’s neurodevelopment. Environ Res. 2022;203:111890. 34418446 10.1016/j.envres.2021.111890

[98] Egalini F, Marinelli L, Rossi M, Motta G, Prencipe N, Rossetto Giaccherino R, et al. Endocrine disrupting chemicals: effects on pituitary, thyroid and adrenal glands. Endocrine. 2022;78:395–405. 35604630 10.1007/s12020-022-03076-x PMC9637063

[99] U.S. Environmental Protection Agency. Consideration of the FQPA Safety Factor and Other Uncertainty Factors in Cumulative Risk Assessment of Chemicals Sharing a Common Mechanism of Toxicity. 2002 Feb.

[100] Ballesteros-Gómez A, Rubio S, Pérez-Bendito D. Analytical methods for the determination of bisphenol A in food. J Chromatogr A. 2009;1216:449–69. 18635192 10.1016/j.chroma.2008.06.037

[101] Warakaulle S, Mohamed H, Ranasinghe M, Shah I, Yanyang X, Chen G, et al. Advancement of milk protein analysis: from determination of total proteins to their identification and quantification by proteomic approaches. J Food Compos Anal. 2024;126:105854.

