# Supplementary material for: Endocrine Disrupting Chemicals in Human Milk: A Systematic Review of Concentrations and Potential Health Implications
Source: Curr Environ Health Rep. 2025 Nov 25;12(1):48. doi: 10.1007/s40572-025-00515-5 (PMC12644229; doi:10.1007/s40572-025-00515-5)
Supplement: Supplementary file 1 — Supplementary Material 1 (PDF 356 KB) [file 40572_2025_515_MOESM1_ESM.pdf]

Supplemental Materials for Endocrine Disrupting Chemicals in Human Milk: A Systematic Review of Concentrations and Potential Health Implications

Table S1: Tabulated data collected for Bisphenols

Compound	Number of papers detected in	# of samples	Weighted Average: Median	Weighted Average: Maximum
Bisphenol A (BPA)	11	353	2.16	14.78
Bisphenol S (BPS)	3	105	0.01	1.12
Bisphenol AF (BPAF)	2	47	0.01	0.51
Bisphenol F (BPF)	1	3	0.15	0.32

Table S2: Tabulated data collected for Polycyclic aromatic hydrocarbons (PAHs)

Compound	Number of papers detected in	# of samples	Weighted Average: Median	Weighted Average: Maximum
Naphthalene	5	218	66.96	134.20
Acenaphthylene	7	350	3.21	15.25
Acenaphthene	7	308	11.86	44.72
Fluorene	8	359	11.73	41.26
Phenanthrene	10	418	29.98	110.11
Anthracene	8	360	6.27	25.91
Fluoranthene	9	296	15.00	62.75
Pyrene	10	414	17.09	50.76
cyclopenta[c,d]pyrene	1	60	0.10	0.43
benz[a]anthracene	5	174	4.25	14.37
Chrysene	7	282	5.67	15.89
5-methylchrysene	1	60	8.29	15.27
benzo[b]fluoranthene	6	230	2.63	15.06
benzo[k]fluoranthene	6	255	1.60	9.35
benzo[j]fluoranthene	2	85	1.61	3.21
benzo[a]pyrene	5	234	0.43	1.54
indeno[1,2,3-cd]pyrene	4	195	1.10	6.13
benzo[g,h,i]perylene	7	247	0.62	7.16
Dibenz[a,h]anthracene	3	194	0.52	18.96
Benzo[b + k]fluoranthene	2	43	21.57	107.38
benzo[c]fluorene	1	38	0.86	4.84
Benzo[b+j]fluoranthene	1	65	0.08	0.32

Table S3: Tabulated data collected for Parabens

Compound	Number of papers detected in	# of samples	Weighted Average: Median	Weighted Average: Maximum
Methylparaben	10	322	0.31	24.72
Ethylparaben	9	310	0.29	11.64
Propylparaben	9	264	0.05	5.09
Butylparaben	6	89	0.00	3.66
Isopropylparaben	1	2	1.58	2.20
Isobutylparaben	1	2	0.52	0.89
Benzylparaben	1	7	0.00	0.16

Table S4: Tabulated data collected for Polybrominated diphenyl ethers (PBDEs)

Compound	Number of papers detected in	# of samples	Weighted Average: Median	Weighted Average: Maximum
PBDE-47	10	862	14.11	574.51
PBDE-99	9	701	2.84	163.05
PBDE-100	10	654	3.25	99.17
PBDE-153	10	926	2.98	91.56
PBDE-154	7	578	0.25	4.69
PBDE-183	7	445	0.07	2.25
PBDE-209	5	437	1.08	4.64
PBDE-15	3	132	0.28	1.07
PBDE-28	7	451	1.37	32.91
PBDE-196	2	25	0.62	1.22
PBDE-197	2	107	0.16	0.44
PBDE-206	2	23	1.62	0.50
PBDE-207	2	107	0.37	0.44
PBDE-49	1	4	0.03	0.11
PBDE-66	2	143	0.00	10.69
PBDE-85	2	201	0.49	26.34
PBDE-138	1	4	0.02	0.04
PBDE-118	1	23	0.06	0.15

Table S5: Tabulated data collected for Polychlorinated biphenyls (PCBs)

Compound	Number of papers detected in	# of samples	Weighted Average: Median	Weighted Average: Maximum
PCB18	1	5	8.53	14.38
PCB28	8	440	2.13	48.03
PCB37	1	4	12.81	37.33
PCB44	1	2	16.93	22.94
PCB49	1	1	6.81	10.45
PCB52	8	406	6.00	341.41
PCB66	1	33	0.95	0.95
PCB70	1	7	9.90	18.89
PCB74	3	140	3.75	5.82
PCB77	3	71	0.06	0.16
PCB81	2	70	0.02	0.02
PCB87	1	8	14.88	31.69
PCB99	2	135	3.81	4.19
PCB101	8	434	3.67	30.55
PCB105	5	175	1.32	2.64
PCB114	2	70	0.10	0.23
PCB118	10	615	11.58	48.95
PCB119	1	5	19.65	58.89
PCB123	3	73	0.26	0.48
PCB126	2	70	0.01	0.02
PCB128	1	9	20.98	52.42
PCB138	8	684	33.66	318.83
PCB138/158	1	35	20.47	0.00
PCB146	2	134	2.20	2.78
PCB151	1	4	15.73	28.67
PCB153	10	672	53.01	458.02
PCB156	4	204	1.39	1.86
PCB157	2	70	0.10	0.26
PCB164/163	1	100	2.87	5.11
PCB167	2	70	0.16	0.50
PCB169	2	70	0.01	0.02
PCB170	3	219	4.00	6.00
PCB177	2	29	2.29	3.11
PCB178	1	30	0.97	0.00
PCB180	9	698	32.98	483.57
PCB182/187	1	100	4.30	7.27
PCB183	2	32	1.50	0.59
PCB187	2	40	4.97	4.32

PCB189	3	72	0.66	0.93
PCB194	3	131	1.08	1.24
PCB196/203	1	33	1.63	0.00
PCB199	2	101	0.92	1.48
PCB201	1	31	1.49	0.00
PCB206	2	116	0.26	0.23
PCB209	1	97	0.10	0.18

Table S6: Tabulated data collected for Organochlorine pesticides (OCPs)

Compound	Number of papers detected in	# of samples	Weighted Average: Median	Weighted Average: Maximum
p,p Dichlorodiphenyldichloroethylene (DDE)	11	1552	1482.92	1929.61
p,p Dichlorodiphenyldichloroethane (DDD)	8	415	89.09	506.69
p,p Dichlorodiphenyltrichloroethane (DDT)	10	1348	215.09	451.64
α-hexachlorocyclohexane (α-HCH)	5	218	0.29	2.68
β-Hexachlorocyclohexane (β-HCH)	8	404	96.71	259.01
γ-Hexachlorocyclohexane (γ-HCH)	7	306	71.77	107.74
Oxychlordane	5	221	1.72	14.20
Cis-chlordane	3	67	0.22	0.92
trans-Nonachlor	3	115	1.22	22.60
Dieldrin	4	240	0.89	228.95
Mirex	2	52	0.26	2.16
δ-Hexachlorocyclohexane (δ-HCH)	2	92	0.25	6.87
Cypermethrin	1	34	1194.40	1443.80
Heptachlor	2	102	0.20	1.78
Heptachlorepoxide	2	99	0.82	3.95
Aldrin	2	98	0.09	1.27
Endrin	2	98	4.19	1.87
Endosulfan-I	2	83	0.07	1.69
Endosulfan-II	2	72	0.02	1.92
Methoxychlor	2	73	0.12	0.61
Pentachlorobenzene	1	47	0.31	2.56
Hexachlorobenzene	2	131	1.01	33.47
Pentachloroanisol	1	47	5.45	0.51
Octachlorostyrene	1	47	0.11	0.41
Trans-chlordane	1	47	0.00	0.06
Cis-heptachlor	1	84	0.00	8.40
Parlar 26 toxaphen	1	84	0.00	1.52
Parlar 50 toxaphen	1	84	0.00	3.10
Chlorpyrifos-ethyl	1	84	0.00	49.59
Endosulfan sulfate	1	47	0.08	0.74
Endrin ketone	1	29	0.02	0.67
Endrin aldehyde	1	38	0.08	2.26
Cis-Chlorantraniliprole	1	43	0.05	0.48
Trans-Chlorantraniliprole	1	28	0.07	1.90
o,p DDD	1	47	0.21	2.88
o,p DDE	2	60	0.71	2.30
o,p DDT	3	216	0.30	7.14

Table S7: Tabulated data collected for Per- and polyfluoroalkyl substances (PFAS) (3 decimal places shown to depict differences between weighted averages for medians and maxima)

Acronym	Full Chemical Name	Number of papers detected in	# of samples	Weighted Average: Median	Weighted Average: Maximum
PFOA	Perfluorooctanoic acid	11	1122	0.044	0.302
PFOS	Perfluorooctane sulfonate	9	928	0.057	0.187
PFHxS	Perfluorohexane sulfonic acid	5	101	0.006	0.051
PFDA	Perfluorodecanoic acid	7	368	0.016	0.186
PFNA	Perfluorononanoic acid	6	276	0.006	0.096
PFBS	Perfluorobutane sulfonic acid	2	30	0.001	0.189
PFHxA	Perfluorohexanoic acid	3	171	0.002	0.257
PFHpA	Perfluoroheptanoic acid	3	134	0.014	0.472
PFUnDA	Perfluoroundecanoic acid	2	35	0.001	0.014
PFDoDA	Perfluorododecanoic acid	2	30	0.001	0.128
PFPeA	Perfluoropentanoic acid	1	118	0.002	0.025
PFTrDA	Perfluorotridecanoic acid	2	90	0.002	0.143
PFPeS	Perfluoropentanesulfonic acid	1	4	0.000	0.002
PFDS	Perfluorodecanesulfonic acid	2	7	0.042	0.050
FTS	Fluorotelomer sulfonic acid	1	7	0.001	0.035
PFHpS	Perfluoroheptanesulfonic acid	1	37	0.001	0.007
PFNS	Perfluorononanesulfonic acid	1	29	0.000	0.001

Appendix A: Fluid adjustment for values given in ng/g lipid within the phthalate chemical class category

1. Lipid content of breastmilk was found to be 3-5% by weight; taking the average of this, we approximated lipid content to be 4% by weight (0.04 g lipid/g breastmilk) [1].
2. Using the density of breastmilk as 1.03g/mL, we were able to convert to a volumetric measurement. The full conversion is shown in the equation below [2].

$$\frac{X\ ng}{g\ lipid} * \frac{0.04 g\ lipid}{g\ breastmilk} * \frac{1.03\ g\ breastmilk}{mL\ breastmilk}$$

This calculation was performed on any value in the phthalate category that was originally measured in ng/g lipid. Although this may not accurately represent the concentration of phthalates in breastmilk in comparison to a direct fluid measurement, this allowed us to compare concentrations across multiple studies to determine the maximum concentrations detected of each phthalate compound.

Table S8: Tabulated data collected for phthalates

Acronym	Full Chemical Name	Number of papers detected in	# of samples	Weighted Average: Median	Weighted Average: Maximum
MEOHP	Mono(2-ethyl-5-oxohexyl) phthalate	3	21	0.06	0.59
MEHHP	Mono(2-ethyl-5-hydroxyhexyl) phthalate	4	45	0.04	0.30
MnBP	Mono-n-butyl phthalate	5	347	2.62	13.99
MiBP	Monoisobutyl phthalate	7	434	4.18	18.11
MEHP	Mono(2-ethylhexyl) phthalate	8	508	2.59	16.78
MEP	Monoethyl phthalate	4	321	0.19	2.25
MECPP	Mono(2-ethyl-5-carboxypentyl) phthalate	2	30	0.06	0.68
MCMHP	Mono[2-(carboxymethyl)hexyl] phthalate	1	19	0.10	0.80
MBzP	Monobenzyl phthalate	4	188	0.02	0.39
DEHP	Di(2-ethylhexyl) phthalate	4	169	5.21	110.63
DnBP	Dibutyl phthalate	2	96	0.64	8.66
DiBP	Diisobutyl phthalate	3	133	8.79	26.79
DcHP	Dicyclohexyl phthalate	2	35	0.00	0.26
DAP	Diallyl phthalate	2	28	0.06	0.61
DEP	Diethyl phthalate	1	8	0.220	1.450
DBP	Dibutyl phthalate	2	29	0.635	8.363
BBzP	Butyl benzyl phthalate	2	68	0.295	4.360
DOP	Dioctyl phthalate	1	10	0.240	11.000
7OH-MMeOP	7-hydroxy-methyl-methyl-octyl phthalate	1	6	1.200	1.500
DiNP	Diisononyl phthalate	1	13	0.000	1.500
MiNP	Mono-isononyl phthalate	1	12	0.000	0.610

Table S9: Papers which noted excess risk for infant exposure to EDCs based on Reference Doses (RfDs) or Total Daily Intakes (TDIs).

Chemical Class	Excess risk noted	Citation
Phthalates	"For risk assessment, the endocrine-related toxicity of the monoester was assumed to be the same as that of its diester form. Median daily intake estimates of phthalates, including both monoester and diester forms, through breast milk consumption ranged between 0.91 and 6.52 μg/kg body weight (bw) for DEHP and between 0.38 and 1.43 μg/kg bw for di-n-butyl phthalate (DnBP). Based on the estimated daily intake, up to 8% of infants exceeded the reference dose of anti-androgenicity (RfD AA) for DEHP, and 6% of infants exceeded the tolerable daily intake(TDI) for DnBP."	[3]
PCBs, OCPs, PBDEs	"When intake values are compared with recent reference doses or minimal risk levels, it becomes evident that mean and/or maximum concentrations of several chemicals are considerably above these levels."	[4]
PCBs, OCPs	"Although the intake of OCs by most infants is below the guideline standards, the intake by some individuals is close to or exceeded these guidelines. This fact suggests that specific individuals, even in the general population, may potentially be at risk of environmental contaminants."	[5]
PCBs, OCPs	"The estimated daily intake (ng/kg body weight/day) of ∑DDTs, dieldrin and nondioxin-like PCBs (∑6PCBs) exceeded the provisional tolerable daily intake (PTDI) in two, six and forty-eight of the nursing infants, respectively, suggesting potential health risks."	[6]
OCPs	"The estimated daily intakes of DDTs by infants indicated that 7 out of 29 of the human milk samples exceeded 20 ng g− 1 day− 1, the tolerable daily intake (TDI) proposed by the Health Canada Guideline in terms of DDTs levels. The high intake of DDTs by infants may be of concern as infants are more susceptible to the adverse effects imposed by various environmental contaminants."	[7]
Parabens	"By incorporating the human equivalent dose and an additional 10-fold margin of safety for infants into the health risk assessment, the 95th percentile hazard quotient of PrP via the ingestion of breastmilk among neonates exceeded 1. For the first time, the results showed that exposure to PrP via breastmilk intake may pose a considerable health risk to urban neonates in southern China."	[8]
PAHs	"Monte Carlo simulations were used to estimate the hazard quotient (HQ) and incremental lifetime cancer risk (ILCR) for infant dietary exposure to PAHs. HQs were below the safe thresholds (HQ = 1) while ILCRs were greater than the reference value equal to 10−6 (mg kg−1day−1)."	[9]
PAHs	"Results showed a high percentage of samples of both breast milk and infant formulas with margin of exposure (MOE) value indicating a potential concern for consumer health."	[10]

PAHs	"However, the estimated margin of exposure (MOE) values of BaP-MOE, PAH2-MOE, PAH4-MOE, and PAH8-MOE were smaller than 10,000 which indicated that there are potential hazard for breastfed infants consuming these human milk samples."	[11]
PCBs	"The exposure of infants by PCDDs/PCDFs and dioxin-like PCBs is still exceeding the daily tolerable intake (TDI) in North West Russia."	[12]
OCPs	"The mean concentration of total DDT at three time points (baseline, midline and endline) were 2.25, 1.68 and 1.32 µg/g milk fat, respectively. The mean concentration of total DDT from the three districts was 1.85 µg/g milk fat which is above the maximum residue limit (MRL = 0.02 µg/g milk fat set by FAO/WHO). The mean ratio of DDT/DDE for the three areas was calculated less than five (< 5) indicates historical DDT use in the study area. The estimated daily intake of infants at the first month of breastfeeding was 11.24 µg/kg-BW/day, above the provisional tolerable daily intake (PTDI) for total DDT set by FAO/WHO, which is 10 µg/kg body weight"	[13]
OCPs	"Although levels of HCH and DDT residues in breast milk samples have decreased significantly, yet estimated daily intake values for DDT are higher than the FAO/WHO permissible tolerable daily intake values for few infants."	[14]
PFAS	"Using milk PFAS concentrations from 6 weeks may thus underestimate both the true EDI of PFAS for newborns and the proportion of infants exposed to PFAS concentrations in milk above the TWI. A recent commentary indicated that the estimated human milk concentrations of PFOS and PFOA in three Canadian and US studies were higher than recommended daily drinking water levels for children.67 While our reported milk concentrations were higher than the drinking water guidance levels, drinking water advisories do not necessarily apply to infants consuming human milk because of the difference in the matrix and unique period of growth that infants undergo between birth and 2 years. Guidance developed specifically for PFAS in human milk is urgently needed to inform public health efforts."	[15]

Table S10: Trends in Compound Depuration

Chemical Class	Trends	Citation
Phthalates	Decrease in phthalate concentration in breastmilk over a 6 month period	[16]
PBDEs, PCBs, and OCPs	"Contrary to earlier research, we found that lipid-adjusted concentrations of polybrominated diphenyl ethers, polychlorinated biphenyls, polychlorinated dibenzo-p-dioxins and furans, and organochlorine pesticides in serum and milk do not consistently decrease during lactation and can increase for some women… our results suggest a more complex relationship for persistent, lipophilic chemicals with the milk/serum relationship dependent on chemical"	[1]
PFAS	The number of PFAS species detected in human milk increased as the lactation time went on from 0.5 to 3 months, and the concentrations of 10 PFAS displayed an increasing trend as the prolongation of lactation time ($p < 0.05$).	[17]
PFAS, PBDEs, and PCBs	After a year of nursing the breast-milk concentrations of PFCs, PBDEs, and PCBs were reduced by 15−94%.	[18]
OCPs, PCBs	Levels of ∑OCPs and ∑PCBs decreased 56 and 30% in Murmansk and 36 and 43% in Arkhangelsk during the study period. The decline of ∑OCPs was significant at both locations ($p < 0.05$–$p < 0.0001$). For ∑PCBs, the decreasing trend was only significant in Arkhangelsk ($p < 0.0001$).	[12]
PFAS	"A one-week increase in the postpartum week of collection was associated with a 3% decrease in milk PFOA concentrations (95% CI: −6, −1%) and 2% decrease in milk PFOS concentrations (95% CI: −4, 1%) (Figure 2; Table 3), although most milk samples (94.8%) were collected before 10 weeks postpartum (Table 1)."[15]	[15]

References

1. LaKind Judy S., Berlin Cheston M., Sjödin Andreas, Turner Wayman, Wang Richard Y., Needham Larry L., et al. Do Human Milk Concentrations of Persistent Organic Chemicals Really Decline During Lactation? Chemical Concentrations During Lactation and Milk/Serum Partitioning. Environ Health Perspect. 2009;117:1625–31.

2. Institute of Medicine. Nutrition During Lactation [Internet]. Washington, DC: The National Academies Press; 1991. Available from: https://nap.nationalacademies.org/catalog/1577/nutrition-during-lactation

3. Kim S, Lee J, Park J, Kim H-J, Cho G, Kim G-H, et al. Concentrations of phthalate metabolites in breast milk in Korea: Estimating exposure to phthalates and potential risks among breast-fed infants. Sci Total Environ. 2015;508:13–9.

4. Schlumpf M, Kypke K, Wittassek M, Angerer J, Mascher H, Mascher D, et al. Exposure patterns of UV filters, fragrances, parabens, phthalates, organochlor pesticides, PBDEs, and PCBs in human milk: Correlation of UV filters with use of cosmetics. Chemosphere. 2010;81:1171–83.

5. Ennaceur S, Gandoura N, Driss MR. Distribution of polychlorinated biphenyls and organochlorine pesticides in human breast milk from various locations in Tunisia: Levels of contamination, influencing factors, and infant risk assessment. Environ Res. 2008;108:86–93.

6. Müller MHB, Polder A, Brynildsrud OB, Karimi M, Lie E, Manyilizu WB, et al. Organochlorine pesticides (OCPs) and polychlorinated biphenyls (PCBs) in human breast milk and associated health risks to nursing infants in Northern Tanzania. Environ Res. 2017;154:425–34.

7. Tsang HL, Wu S, Leung CKM, Tao S, Wong MH. Body burden of POPs of Hong Kong residents, based on human milk, maternal and cord serum. Environ Int. 2011;37:142–51.

8. Zhang D, Xiao J, Xiao Q, Chen Y, Li X, Zheng Q, et al. Infant exposure to parabens, triclosan, and triclocarban via breastfeeding and formula supplementing in southern China. Sci Total Environ. 2023;858:159820.

9. Torres-Moreno C, Puente-DelaCruz L, Codling G, Villa AL, Cobo M, Klanova J, et al. Polycyclic aromatic hydrocarbons (PAHs) in human breast milk from Colombia: Spatial occurrence, sources and probabilistic risk assessment. Environ Res. 2022;204:111981.

10. Santonicola S, De Felice A, Cobellis L, Passariello N, Peluso A, Murru N, et al. Comparative study on the occurrence of polycyclic aromatic hydrocarbons in breast milk and infant formula and risk assessment. Chemosphere. 2017;175:383–90.

11. Wang L, Liu A, Zhao Y, Mu X, Huang T, Gao H, et al. The levels of polycyclic aromatic hydrocarbons (PAHs) in human milk and exposure risk to breastfed infants in petrochemical industrialized Lanzhou Valley, Northwest China. Environ Sci Pollut Res. 2018;25:16754–66.

12. Polder A, Gabrielsen GW, Odland JØ, Savinova TN, Tkachev✠ A, Løken KB, et al. Spatial and temporal changes of chlorinated pesticides, PCBs, dioxins (PCDDs/PCDFs) and brominated flame retardants in human breast milk from Northern Russia. Sci Total Environ. 2008;391:41–54.

13. Mekonen S, Ambelu A, Wondafrash M, Kolsteren P, Spanoghe P. Exposure of infants to organochlorine pesticides from breast milk consumption in southwestern Ethiopia. Sci Rep. 2021;11:22053.

14. Bedi JS, Gill JPS, Aulakh RS, Kaur P, Sharma A, Pooni PA. Pesticide residues in human breast milk: Risk assessment for infants from Punjab, India. Sci Total Environ. 2013;463–464:720–6.

15. Criswell RL, Wang Y, Christensen B, Botelho JC, Calafat AM, Peterson LA, et al. Concentrations of Per- and Polyfluoroalkyl Substances in Paired Maternal Plasma and Human Milk in the New Hampshire Birth Cohort. Environ Sci Technol. 2022;57:463–72.

16. Hung S-C, Lin T-I, Suen J-L, Liu H-K, Wu P-L, Wu C-Y, et al. Phthalate Exposure Pattern in Breast Milk within a Six-Month Postpartum Time in Southern Taiwan. Int J Environ Res Public Health. 2021;18.

17. Zhang X, Zhou X, Chen H, Gao X, Zhou Y, Lee HK, et al. Changes in Concentrations of Polyfluoroalkyl Substances in Human Milk Over Lactation Time and Effects of Maternal Exposure via Analysis of Matched Samples. Environ Sci Technol. 2024;58:4115–26.

18. Thomsen C, Haug LS, Stigum H, Frøshaug M, Broadwell SL, Becher G. Changes in Concentrations of Perfluorinated Compounds, Polybrominated Diphenyl Ethers, and Polychlorinated Biphenyls in Norwegian Breast-Milk during Twelve Months of Lactation. Environ Sci Technol. 2010;44:9550–6.
